# Rewiring of auxin and MAPK signaling is associated with contrasting shoestring and fern-like manifestations in ToBRFV-A134T-infected tomato

**DOI:** 10.3389/fpls.2026.1828330

**Published:** 2026-06-30

**Authors:** Ori Molad, Elisheva Smith, Diana Leibman, Noa Sela, Meital Reches, Aviv Dombrovsky

**Affiliations:** 1 Department of Plant Pathology and Weed Research, Agricultural Research Organization (ARO), The Volcani Center, Rishon LeZion, Israel; 2 Institute of Chemistry and The Center for Nanoscience and Nanotechnology, The Hebrew University of Jerusalem, Jerusalem, Israel; 3 Bioinformatics Unit, Agricultural Research Organization (ARO), The Volcani Center, Rishon LeZion, Israel

**Keywords:** disease symptom, RNA seq analysis, tobamovirus, ToBRFV, tomato, auxin, MAP kinase

## Abstract

**Introduction:**

Tomato brown rugose fruit virus (ToBRFV) is a rapidly spreading plant pathogen that threatens tomato cultivation worldwide, leading to economic losses. ToBRFV-A134T, a new isolate, induces a subset of plant responses characterized by unique severe shoestring-like and fern-like manifestations. We aimed to investigate the differential host response of *Tm-2^2^*-resistant tomato plants toward ToBRFV-A134T versus the wildtype isolate (ToBRFV-WT) to understand the mechanisms underlying these distinct manifestations.

**Methods:**

We performed symptomatology analysis to follow the temporal development of shoestring and fern-like symptoms in ToBRFV-A134T-infected tomato plants. Comparative transcriptomic analysis of RNA-seq data from three biological replicates per leaf manifestation, sampled at 38 days post-inoculation, was conducted to identify differentially expressed genes and enriched pathways. In parallel, ToBRFV-WT-infected tomato plants were treated with exogenous auxin to test whether auxin perturbation could recapitulate ToBRFV-A134T-associated symptoms.

**Results:**

Shoestring symptoms consistently preceded fern-like symptoms in ToBRFV-A134T-infected plants and progressed to a mixture of manifestations at late disease stages. ToBRFV-A134T-induced shoestring symptoms were associated with a distinct subset of auxin-responsive genes and enrichment of the MAP kinase pathway. Salicylic acid-mediated defense was suppressed in shoestring leaves but remained active in fern-like leaves, as indicated by downregulation versus upregulation of Pathogenesis related-1 (PR-1) genes, respectively. Despite equivalent ToBRFV accumulation across infected phenotypes, fern-like leaves showed a transcriptional profile highly similar to that of healthy, non-infected controls. Exogenous auxin treatments of ToBRFV-WT-infected plants increased the occurrence of shoestring symptoms and recapitulated the unique fern-like manifestations induced by ToBRFV-A134T.

**Discussion:**

These findings indicate that distinct rewiring of auxin signaling, MAPK signaling, and salicylic acid-mediated defense is associated with divergent foliar manifestations of ToBRFV disease in *Tm-2^2^*-resistant tomato plants. Auxin emerges as a key determinant of symptom identity, linking hormonal crosstalk to the contrasting shoestring and fern-like outcomes observed for the ToBRFV-A134T isolate.

## Introduction

Tobamoviruses are seed-borne and soil-borne, mechanically transmitted viruses that infect a wide range of cultivars (Broadbent, 1965a; Broadbent et al., 1965; Broadbent, 1965b). Tobamoviruses are single-stranded, positive-sense RNA viruses (+ssRNA) of ~6.4 kb genome encoding four principal viral proteins: two comprising the RNA-dependent RNA polymerase (RdRP); a ~30 kDa movement protein (MP); and a ~17 kDa coat protein (CP) (Goelet et al., 1982). The *Tobamovirus* genus (Lefkowitz et al., 2018) includes the tobacco mosaic virus (TMV) (Mayer, 1886), the tomato mosaic virus (ToMV) (Broadbent, 1976) and the newly identified tomato mottle mosaic virus (ToMMV) (Ambrós et al., 2017; Webster et al., 2014). These tomato-infecting viruses could not overcome the *Tm-2^2^* resistance gene (Lanfermeijer et al., 2004, 2003; Nagai et al., 2019; Tettey et al., 2023), introgressed into cultivated tomato plants from the wild *Solanum peruvianum* plant (Lanfermeijer et al., 2003). Tomato brown rugose fruit virus (ToBRFV) is a more recently identified tobamovirus (Salem et al., 2016) that has overcome *Tm-2^2^* resistance in tomato plants (Luria et al., 2017). Severe disease symptoms caused by ToBRFV wild-type (WT) infection of susceptible tomato plants include yellow spotted or brown rugose fruits as well as mottling or mosaic symptoms on leaves (Luria et al., 2017; Salem et al., 2016), occasionally associated with shoestring-like symptoms characteristic of ToMV and ToMMV-infected susceptible tomato plants (Andrade, 1981; Klap et al., 2020; Tettey et al., 2023).

The shoestring-like syndrome in tomato plants has been attributed to upregulation of auxin response factors (ARFs) ARF3 and ARF4 that regulate auxin-responsive genes (Yifhar et al., 2012). Auxin signaling is commonly engaged by plant pathogens upon infection (Manulis et al., 1998; Wang et al., 2007). Auxin has been shown to benefit plant virus infection, specifically via auxin signaling reprogramming in TMV-infected tomato plants. Cytoplasmic sequestration of Aux/Indole acetic acid (IAA) by binding to TMV replicase (Padmanabhan et al., 2008, 2006, 2005) has accelerated expression of auxin responsive genes and enhanced cell-to-cell viral movement and phloem uploading (Collum et al., 2016). The function of TMV replicase, together with the MP in alteration of plasmodesmata conductivity and cell-to-cell movement has been documented (Guenoune-Gelbart et al., 2008). Consistent with this, it has been shown that a highly active auxin signaling compromised plant resistance (Wang et al., 2007). In studies of hormonal interactions, inhibition of sensitivity to auxin was apparently essential for the salicylic acid (SA) associated plant defense response. Over-accumulation of SA counteracted auxin signaling, leading to enhanced plant disease-resistance (Wang et al., 2007). Importantly, SA participates in plant response to TMV: negative modulation of SA was found required for TMV systemic movement in infected *Arabidopsis thaliana* plants (Venturuzzi et al., 2021). Upstream of these hormonal interactions, the Mitogen-activated protein kinase (MAPK) signaling pathway serves as a central regulator of diverse immune responses (Meng and Zhang, 2013), participates as well in suppression of auxin signaling (Kovtun et al., 1998; Zhang and Klessig, 2001), whereas ethylene (ET) signaling is promoted by the pathway (Meng and Zhang, 2013; Taj et al., 2010). ET signaling enhanced response to SA, potentiating expression of pathogenesis-related protein 1 (PR-1) (De Vos et al., 2006; Lawton et al., 1994). Interestingly, in TMV-infected tobacco plants, an increase in ET perception was shown to be essential for the onset of SA-induced systemic acquired resistance (SAR) against the virus (Verberne et al., 2003).

We recently identified a mutant of ToBRFV that contained a single nonsynonymous A134T amino-acid substitution in the MP that causes unique symptoms in tomato plants (Molad et al., 2025). ToBRFV-A134T-infected tomato plants showed severe shoestring-like symptoms with an occasional development of fern-like symptoms, the latter showing a seemingly compensatory increase in leaf area (Molad et al., 2025). These observations raised the hypothesis that the A134T mutation in the ToBRFV MP triggers a distinct rewiring of hormonal and defense pathways in tomato plants, thereby inducing unique foliar manifestations. The present study focuses on characterizing the transcriptional states associated with distinct foliar symptom manifestations induced by ToBRFV-A134T infection at a defined late disease stage and equivalent leaf developmental age, building on the temporal observations of early symptom progression. We combine high-throughput sequencing (HTS) analyses and targeted RT-qPCR of selected genes to compare ToBRFV-WT and ToBRFV-A134T-induced symptoms, and to dissect the transcriptional programs associated with mosaic, shoestring and fern-like manifestations. We show that each symptom is associated with a distinct transcriptional profile, and that ToBRFV-A134T-induced shoestring symptoms are associated with rewiring of auxin signaling, along with enrichment of the MAPK pathway.

## Materials and methods

### Plant material and growth conditions for visualization of disease stages and total RNA extractions during ToBRFV-WT and ToBRFV-A134T-infection

Tomato plants cv. Ikram, harboring *Tm-2^2^* resistance, were grown in temperature- controlled growth chambers, at 25 ± 2 °C. 39 plants, at the 5–7 true leaf stage, were subjected to sap-mechanical inoculations, prepared from ToBRFV-A134T-infected *Tm-2^2^*-resistant tomato plants (~1 g leaves/10 mL 0.01M Na-Phosphate buffer, pH=7.0), confirmed by viral RNA extraction and Sanger sequencing to contain the mutant. Leaf manifestations were monitored over 38 days following the inoculation of ToBRFV-WT-infected plants, and either severe shoestring-like symptoms or fern-like symptoms in ToBRFV-A134T-infected plants. For each symptom and for the healthy control plants, leaves from a total of 144 were sampled from the third leaf beneath the meristem. For each iteration, 3 leaves from three different plants were sampled and subjected to total-RNA extractions followed by high-throughput sequencing (HTS) and/or RT-qPCR analysis (see below).

### Viral-RNA extractions and RT-PCR amplifications for ToBRFV isolate identification

Tomato plant leaves (~0.6 g/mL), were subjected to RNA extraction using Bioreba extraction buffer (Bioreba, Reinach, Switzerland). Viral RNA extraction was performed using Viral Nucleic Acid Extraction Kit II (Geneaid Biotech Ltd. Taiwan). Using a commercial Verso™ cDNA Synthesis Kit (Thermo Fisher Scientific, MA, USA), the complementary DNA (cDNA) was generated. The cDNA served as a template for polymerase chain reactions (PCR) using a commercial Rapid Taq Master Mix kit (Vazyme, Nanjing, PRC). For mutation verification, we used a primer pair designed for ToBRFV MP (**Table 1** – Set 1). The 683 bp amplicons were Sanger sequenced (HyLabs, Rehovot, Israel).

**Table 1:** List of genes and primers for polymerase chain reactions.

Set	Name	Description	Accession^a^	Amplicon size (bp)	Direction	Sequence
1	ToBRFV-WT	Tomato brown rugose fruit virus-Israeli isolate ToBRFV-IL, complete genome^b^	KX619418	683	(5’-3’)	GGAGAGAGCGGACGAGGCAA
	ToBRFV-A134T	Tomato brown rudgose fruit virus isolate A134T, complete genome^b^	PP681638		(3’-5’)	ACAGGTTTCCACACTTCGCT
2	*SAUR1*	Small auxin up-regulated RNA1	Solyc01g091030	109	(5’-3’)	AAGGCTATTTGCAGCAAGGG
					(3’-5’)	AAACCGCCAAGTGACCTTTC
3	*MPK3*	Mitogen-activated protein kinase 3	Solyc06g005170	86	(5’-3’)	CAGCCTTCAATTTGCAGCCA
					(3’-5’)	AACACAAGCTAGCCCGAACA
4	*ERF.D7*	Ethylene responsive transcription factor D7	Solyc03g118190	100	(5’-3’)	GAGTTCGTCAAAGGCCATGG
					(3’-5’)	GCACCTTCCGCTGTTTCAAA
5	*ABA4*	Abscisic acid (Aba)-deficient 4	Solyc02g086050	120	(5’-3’)	CTGCTTGGATTCACCTATTGGC
					(3’-5’)	TGGGGCAAAATAGCAAGCAG
6	*CHI-like*	Chalcone-flavanone isomerase family protein	Solyc02g083890	91	(5’-3’)	CTCCCATGGGTCTTCTTGCC
					(3’-5’)	ACAGGGGATGCAGTAACAGC
7	*GAI*	GAI	Solyc11g011260	99	(5’-3’)	TTAATGGCGTGTGCTGAAGC
					(3’-5’)	AGCACCAGATTGTGAAACCG
8	*PR1-1*	Pathogenesis-related protein PR-1	Solyc07g006710	136	(5’-3’)	TCAATGCCCTCACTCAACAAC
					(3’-5’)	GGTTTTAGCCTAAGCATTGAACG
9	*TIP41*	TIP41-like protein	Solyc10g049850	96	(5’-3’)	GCTGCGTTTCTGGCTTAGG
					(3’-5’)	ATGGAGTTTTTGAGTCTTCTGC
10	ToBRFV-CP	Tomato brown rugose fruit virus	KX619418/ PP681638	159	(5’-3’)	CACAATCGCAACTCCATCGC
					(3’-5’)	ACAGGTTTCCACACTTCGCT

^a^ Accession numbers correspond to ITAG4.1 annotations. ^b^ NCBI accession.

### Total RNA extraction followed by HTS analyses using Illumina HiSeq platform

Tomato plant leaves (~0.1 g/mL) of each manifestation stage were sampled at 38 days post inoculation (dpi) and subjected to total RNA extraction using Ribospin™ Plant kit (GeneAll, Seoul, South Korea). For each manifestation, three replicates pooled from three plants were sampled for a total of nine plants. RNA quality and quantity were determined using an ND1000 spectrophotometer (Nano Drop Technologies Inc., USA) and RNA integrity was later assessed using an Agilent TapeStation system according to the sequencing provider’s quality thresholds. cDNA libraries were prepared for poly-A RNA-seq, which enriches for host mRNAs and is widely used in plant virus-host interaction studies, and sequenced as single-end 150 bp reads on an Illumina HiSeq 2500 platform (Weizmann Institute of Science, Rehovot, Israel) (Conesa et al., 2016). Clean reads were obtained by removing the adaptor and low-quality sequences from Hiseq raw reads using Trimmomatic software version 0.39 (Bolger et al., 2014). On average, each library contained 30 million reads (range 16-50). The tomato genome version SL4.0 and ITAG4.0 annotation were downloaded from Sol Genomics Network, (https://solgenomics.net/organism/Solanum_lycopersicum/genome) (Fernandez-Pozo et al., 2015). RNA-seq data generated in this study have been deposited in the NCBI Sequence Read Archive (SRA) under BioProject accession PRJNA1431405. For gene expression analyses clean reads were mapped to tomato genome using STAR version v2.7.11 (Dobin et al., 2013) and then we used HTSeq suit for gene quantification (Anders et al., 2015). We calculated gene differential expression with DESeq2 R package (Love et al., 2014), with a threshold of FDR < 0.05 and log2fold change greater than 1 or smaller than -1. KEGG pathway enrichment analysis was performed using the KOBAS web server (Bu et al., 2021), applying a hypergeometric test with Benjamini-Hochberg FDR correction, using pathway-specific background gene sets derived from the *S. lycopersicum* KEGG annotation. GO term enrichment analysis was performed using the g:Profiler website (Kolberg et al., 2023), querying GO Biological Process, Molecular Function, and Cellular Component ontologies, with the background defined as all GO-annotated tomato genes in the database (effective domain size: ~13,614). Enriched terms and pathways were considered significant at FDR < 0.05 (-log10(FDR)≥1.3). The whole-genome expression heatmap was generated in Python using Seaborn’s clustermap, with Ward’s linkage and Euclidean distance for hierarchical clustering of genes and samples, and samples annotated by condition using colored column bars (Waskom, 2021). MA plots were generated in Python using matplotlib and Seaborn (Hunter, 2007). Venn diagrams summarizing overlaps between differentially expressed gene sets were computed in Python using the venny4py package (Yergaliyev, 2026). Clustered heatmaps of hormone-related pathways were visualized in R using the ComplexHeatmap package (Gu, 2022).

### Total RNA extractions and quantitative RT-PCR

In each of 3 experiments, tomato plant leaves (~0.1 g/mL) from 9 plants for each symptom manifestation stage were subjected to total RNA extraction using a TRI Reagent kit (MRC, Inc., Cincinnati, OH, USA). cDNA synthesis was performed with Random Hexamers, using Verso™ cDNA Kit (Thermo Fisher Scientific, Epsom, UK). RT-qPCR was conducted primarily as described before (Klap et al., 2020), using the StepOnePlus™ (Applied Biosystems, Fisher Scientific Company, Ottawa, Ontario). All reactions were performed in triplicate. cDNA transcribed from 100 ng RNA was subjected to RT-qPCR in a 15 µl reaction mixture containing 4 µl 1:5 diluted cDNA, 3 pmols of each primer, and 7.5 µl AzuraView™ GreenFast qPCR Blue Mix HR (Azura Genomics Inc., MA, USA). For the tested genes, primers were designed using Primer3Plus (EMBL). The tested tomato genes were - mitogen-activated protein kinase 3 (MPK3, Solyc06g005170), Small auxin up-regulated RNA1 (SAUR1, Solyc01g091030), Ethylene responsive transcription factor D7 (ERF.D7, Solyc03g118190), Abscisic acid deficient 4 (ABA4, Solyc02g086050), Chalcone-flavanone isomerase family protein (CHI-like, Solyc02g083890), Gibberellic acid insensitive (GAI, Solyc11g011260), and Pathogenesis-related protein PR-1 (PR1-1, Solyc07g006710), using primer sets 2-8 (**Table 1**). *SlTIP41* (TIP41-like protein, Solyc10g049850) endogenous gene served as a reference gene (Lacerda et al., 2015) using primer set 9. ToBRFV accumulation was quantified using primer set 10, designed for the viral coat protein. For each test, ΔCt was calculated by subtracting the Ct of the endogenous gene from the Ct of the tested gene. For calculations of tomato plant gene-expression, calculation of ΔΔCt was conducted by subtracting the mean ΔCt of the analyzed gene in uninfected healthy control plants from ΔCt of the analyzed gene in virus-infected samples. Relative gene expression was estimated using the 2^−ΔΔCt^ method relative gene expression (Livak and Schmittgen, 2001) and presented as standard error of the mean (SEM). Calculated results of the expressed genes in the virus-infected plants compared to uninfected healthy control plants were subjected to a t-Test, two-sample assuming unequal variances, p ≤ 0.05 (Yuan et al., 2006).

### Recapitulation of disease manifestation of ToBRFV-A134T-infected tomato plants by application of a synthetic auxin to ToBRFV-WT-infected plants

Tomato plants cv. Ikram were grown in temperature-controlled growth chambers kept at 25 ± 2 °C, across five separate experiments. At the 2nd true leaf stage, a synthetic auxin, 1-naphthaleneacetic acid (NAA) solution (40 ppm, 0.04 mg/mL double-distilled water (DDW)) was sprayed to cover the entire foliage surface. One day later, plants were subjected to sap-mechanical inoculations, prepared from either ToBRFV-WT or ToBRFV-A134T-infected *Tm-2^2^*-resistant tomato plants (~1 g leaves/10 mL 0.01M Na-Phosphate buffer, pH=7.0). The healthy control plants were grown under similar conditions and underwent identical NAA treatments. In all five experiments, a second NAA treatment was applied upon the first manifestation of disease symptoms. In a parallel experiment, ToBRFV-WT-infected plants and ToBRFV-A134T-infected plants were mock treated by spraying DDW, for comparison of disease symptoms. NAA-treated ToBRFV-WT-infected plants exhibiting severe shoestring and fern-like symptomatic leaves were sampled for viral RNA extraction to confirm the isolate identity using Sanger sequencing.

## Results

### ToBRFV-A134T-induced severe shoestring-like symptoms in *Tm-2^2^*-resistant tomato plants preceded the appearance of fern-like symptoms

**Figure 1:**
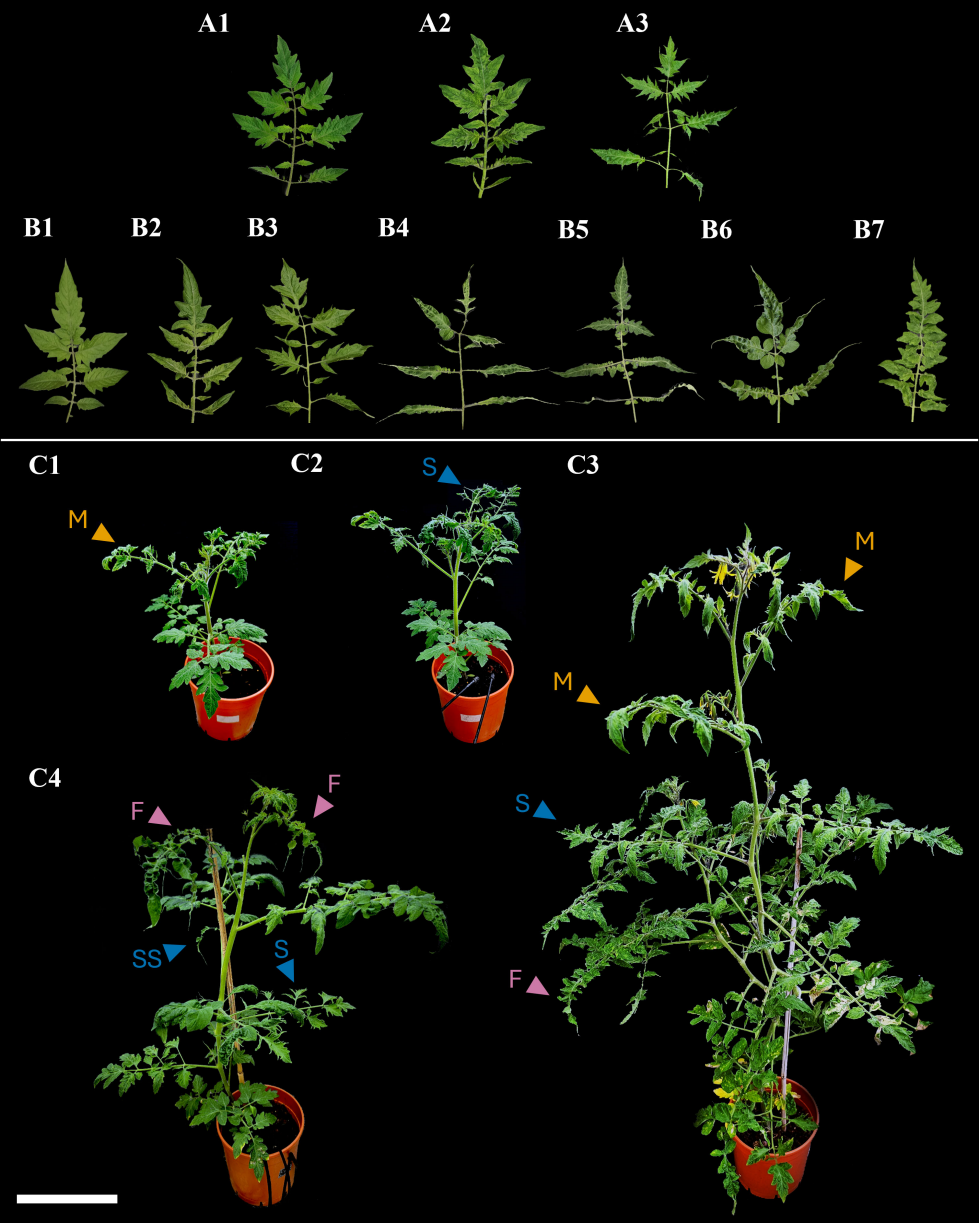
Symptom development in ToBRFV-WT-infected and ToBRFV-A134T-infected tomato plants. **(A1–3)** Symptoms in ToBRFV-WT-infected tomato plants. **(A1)**. An asymptomatic leaf; **(A2)**. Mosaic symptoms; **(A3)** Mosaic and mild shoestring-like symptoms. **(B1–7)** Symptoms in ToBRFV-A134T-infected tomato plants. **(B1)** an asymptomatic leaf; **(B2)** mosaic symptoms; **(B3)** mosaic and mild shoestring-like symptoms; **(B4)** severe shoestring-like symptoms; **(B5)** fern-like symptoms above severe shoestring-like symptoms; **(B6–7)** fern-like symptoms; **(C1–4)** A full plant view of ToBRFV-A134T-infected tomato plants. Symptoms are indicated in arrows and initials – orange (M - mosaic), blue (S - shoestring) and pink (F - fern-like). **(C1)** a ToBRFV-A134T-infected tomato plant at 14 dpi; **(C2)** a ToBRFV-A134T -infected at 17 dpi; **(C3)** a ToBRFV-A134T -infected tomato plant at 24 dpi; **(C4)** a ToBRFV-A134T-infected tomato plant at 47 dpi exhibiting all symptom manifestations. Scale bar for **(C1–4)** is 0.2 m.

**Figure 2:**
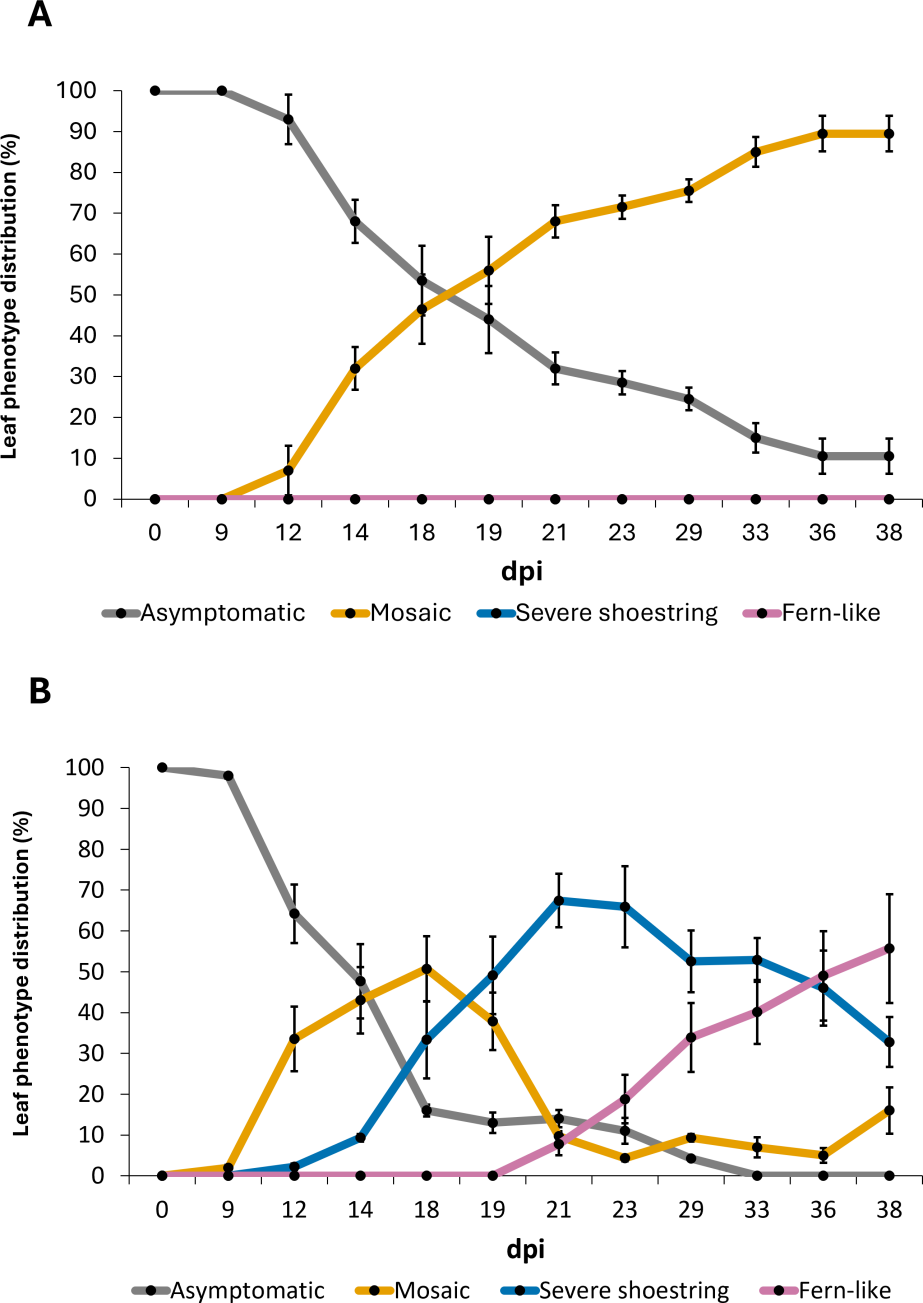
Temporal phenotypic appearance in the five youngest leaves of ToBRFV-infected tomato plants. **(A)** ToBRFV-WT-infected tomato plants; **(B)** ToBRFV-A134T-infected tomato plants.

We previously described the effect of the ToBRFV-A134T mutant on symptom manifestation and development in infected tomato plants, which at advanced infection stages exhibited severe shoestring-like and fern-like symptoms (Molad et al., 2025). The latter symptoms showed a characteristic increase in leaf surface area. At early stages of disease development in ToBRFV-A134T-infected tomato plants, ARF3, characteristic of active auxin signaling during the shoestring-like symptoms, was upregulated. In a detailed timeline of manifestation follow-up, ToBRFV-A134T-infected *Tm-2^2^*-resistant tomato plants showed that the severe shoestring-like symptoms preceded the appearance of the fern-like symptoms (**Figure 1**). In comparison, ToBRFV-WT-infected tomato plants exhibited mosaic and mottling of leaves, with occasional mild shoestring-like symptoms as the most severe phenotype (**Figure 1A**). In ToBRFV-A134T-infected tomato plants, mosaic symptoms were followed by severe shoestring manifestations, which subsequently turned into fern-like leaves (**Figures 1B1–7**). At later stages of infection, the plants’ phenotype showed a combined manifestation of mosaic leaves as well as severe shoestring-like and fern-like leaves (**Figures 1C1–4**). The percentage and manifestation timeline of each of the symptoms over 38 days of growth (25 ± 2 °C) post ToBRFV inoculation are shown in **Figure 2**. As early as 12 dpi, mosaic symptoms emerge similarly in ToBRFV-WT-infected and A134T-infected tomato plants (**Figures 2A, B**). However, starting as early as 14 dpi, severe shoestring symptoms emerge, followed by fern-like symptoms as early as 21 dpi (**Figure 2B**). Although severe shoestring and fern-like leaves become the predominant phenotypes, young leaves with mosaic symptoms reemerge, resulting in a mixture of phenotypes in both young and older leaves (**Figure 2B**). This mixture of symptoms in ToBRFV-A134T-infected tomato plants was retained at least until 105 dpi (**Supplementary Figure 1**).

### Application of synthetic auxin on ToBRFV-WT-infected tomato plants recapitulated ToBRFV-A134T-associated severe shoestring and fern-like manifestations

**Figure 3:**
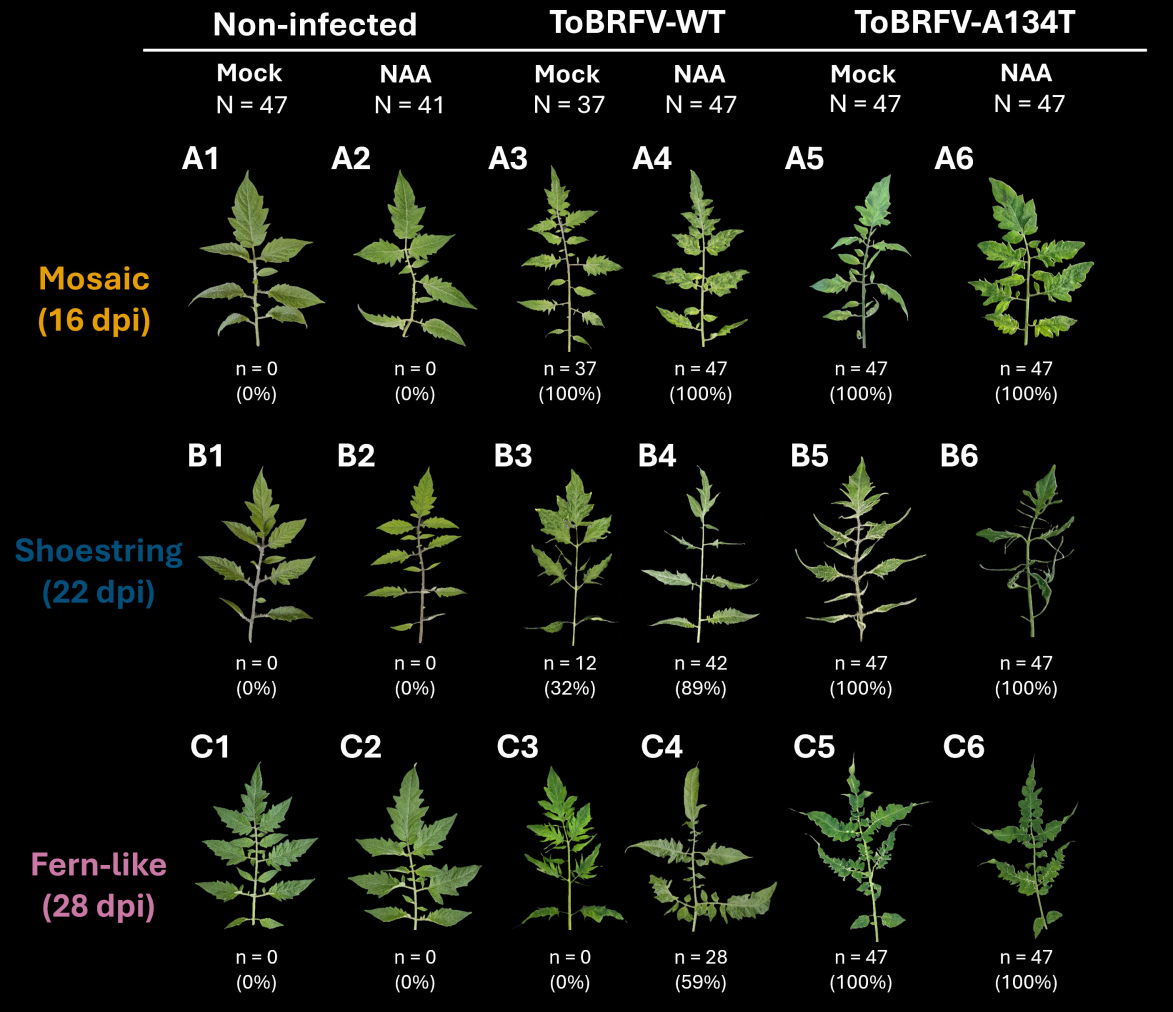
Symptom development in ToBRFV-infected NAA-treated tomato plants. Representative leaves from tomato plants at 16, 22, and 28 days post inoculation (dpi) are arranged in a matrix according to time point (rows) and treatment/inoculation combination (columns). The top row **(A1–A6)** shows leaves at 16 dpi, when mosaic symptoms are predominant; the middle row **(B1–B6)** shows leaves at 22 dpi, when shoestring symptoms are predominant; and the bottom row **(C1–C6)** shows leaves at 28 dpi, when fern-like symptoms are predominant. Columns correspond to non-infected mock-treated plants **(A1, B1, C1)**, non-infected NAA-treated plants **(A2, B2, C2)**, ToBRFV-WT-infected mock-treated plants **(A3, B3, C3)**, ToBRFV-WT-infected NAA-treated plants (a4, b4, c4), ToBRFV-A134T-infected mock-treated plants **(A5, B5, C5)**, and ToBRFV-A134T-infected NAA-treated plants **(A6, B6, C6)**. At the top of each column, N indicates the total number of plants in that specific treatment/inoculation group across all experiments. Below each leaf, the first line (n) indicates the number of plants exhibiting the depicted phenotype out of the total plants tested for that treatment and time point, and the second line shows the corresponding percentage of plants with that phenotype. Data are pooled from three independent experiments; the total number of plants per treatment/inoculation group is indicated as N at the top of each column.

We tested the effect of exogenous auxin on the disease manifestations of ToBRFV-infected tomato plants by treating *Tm-2^2^*-resistant tomato plants with the synthetic auxin NAA, followed by infection with either ToBRFV-WT or ToBRFV-A134T one day later, while non-infected plants served as control. Plants were sprayed with NAA for a second time between 16 and 18 dpi, concurrent with the emergence of mosaic and mottling symptoms (**Figure 3A**). Shoestring-like symptoms appeared between 21 and 22 dpi, followed by fern-like symptoms between 27 and 31 dpi (**Figures 3B, C**). We found that NAA-treated, ToBRFV-WT-infected tomato plants recapitulated the progression of symptoms characteristic of ToBRFV-A134T-infected plants (**Figures 3A4, B4, C4**). Overall, 89% of NAA−treated, ToBRFV−WT−infected plants exhibited shoestring−like symptoms compared to 32% of mock−treated ToBRFV−WT−infected plants (**Figures 3B3, 4**). Additionally, 59% of ToBRFV-WT-infected, NAA-treated tomato plants exhibited severe fern-like symptoms compared to 0% of the mock-treated plants (**Figures 3C3, 4**). In the mock-treated ToBRFV-A134T-infected plants, all 47 plants exhibited severe shoestring-like leaf symptoms followed by fern-like leaf manifestation. NAA treatment of healthy control plants did not induce any foliar manifestations that resembled disease symptoms. Fisher’s exact test confirmed that NAA treatment significantly increased the incidence of both shoestring (OR = 17.5, p < 0.0001) and fern-like symptoms (p < 0.0001) compared to mock-treated ToBRFV-WT-infected plants. In the recapitulation experiment, the fern-like leaves emerged following the manifestation of the shoestring-like symptoms. Using Sanger sequencing we confirmed the exclusive presence of ToBRFV-WT in the NAA-treated plants, showing the characteristic symptoms of ToBRFV-A134T-infected plants.

### Gene expression patterns of fern-like symptomatic ToBRFV-A134T-infected tomato plants and healthy control plants were highly similar

**Figure 4:**
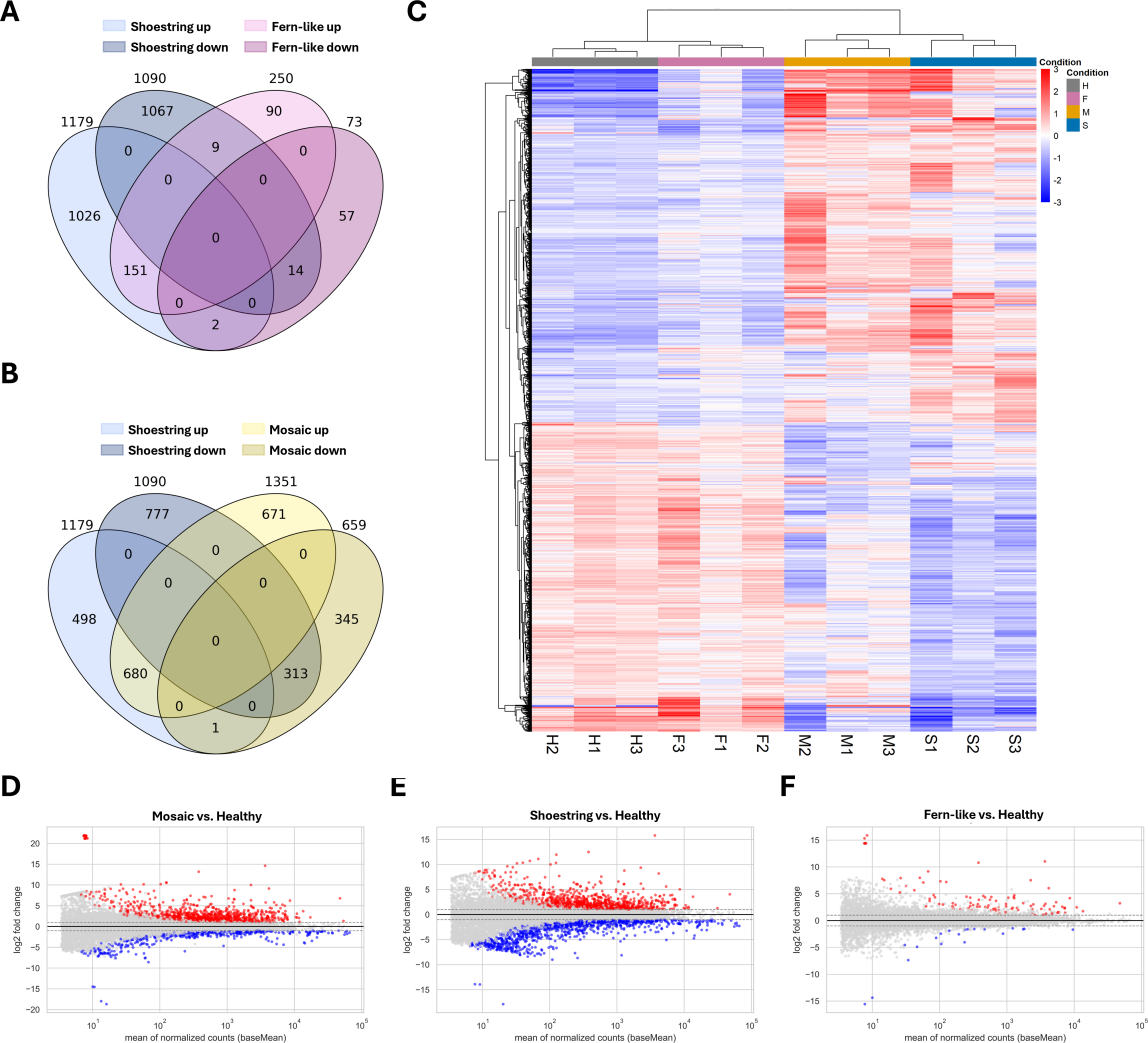
High throughput sequencing analyses of ToBRFV-infected tomato plants. **(A)** Venn diagram comparison of Shoestring upregulated and downregulated genes with Fern-like upregulated and downregulated genes; **(B)** Venn diagram comparison of Shoestring upregulated and downregulated genes with Mosaic upregulated and downregulated genes; **(C)** Heatmap visualization showing a hierarchical clustering of differentially expressed gene analysis. H, Healthy; F, Fern-like; S, Shoestring; M, Mosaic. Color scale represents log2fold change. **(D–F)** MA plot visualizations of differentially expressed genes between Mosaic, Shoestring and Fern-like compared to Healthy. Red dots indicate up-regulated differentially expressed genes (DEGs), blue dots indicate down-regulated DEGs, gray dots indicate non-DEGs.

To gain insight into the molecular mechanisms of ToBRFV-A134T-induced manifestations, ToBRFV-A134T-infected *Tm-2^2^*-resistant tomato plants showing severe shoestring symptoms (Shoestring) and fern-like (Fern-like) symptoms as well as ToBRFV-WT-infected plants showing mosaic symptoms (Mosaic) were sampled at 38 dpi and subjected to HTS analyses of differentially expressed genes compared to healthy non-infected controls (Healthy). These terms are capitalized when referring to RNA−seq sample groups. Annotated DEGs of the three symptomatic plant groups, compared to the healthy control, were analyzed for statistically significant log2fold changes (**Supplementary Table 1** – DEGs lists). Venn diagram analysis of DEGs revealed that the Shoestring and Fern−like groups shared 151 (12%) of upregulated DEGs and 14 (1%) of downregulated DEGs relative to the union of each pair of DEG sets (**Figure 4A**). By comparison, the Shoestring and Mosaic groups shared 680 (37%) of upregulated DEGs and 313 (22%) of downregulated DEGs (**Figure 4B**). The results of the differential expression analysis were visualized using a hierarchical clustering heatmap, showing four distinct clades corresponding to the four distinct phenotypes (**Figure 4C**). Expression profiles indicated that the Healthy samples co-clustered with the Fern-like samples, whereas the Mosaic samples co-clustered with Shoestring samples. The co-clustering of Healthy and Fern-like samples, as well as Mosaic and Shoestring samples, was further supported by principal component analysis (PCA) results (**Supplementary Figure 2**). Overall, the Shoestring clade showed the highest transcriptional distance from the Healthy control samples. (**Figure 4C**). Similarly, MA plot visualization of gene expression results showed that the Shoestring group shared a high fraction of the expressed genes with the Mosaic group (**Figure 4D**). Importantly, most of the DEGs in the Fern-like group and the Healthy group cluster close to the zero line on the Y-axis indicating a highly similar expression pattern of the two groups (**Figure 4D**) (McDermaid et al., 2019).

### Gene ontology enrichment analysis revealed contrasting photosynthetic suppression and lipid−mediated defense in Shoestring and Fern-like leaves

Gene ontology (GO) enrichment analysis of DEGs in the three phenotypic groups showed that Shoestring and Mosaic leaves shared strong enrichment of downregulated GO terms associated with photosynthesis and chloroplast function, including the Biological Process (BP) terms ‘photosynthesis, light reaction’ (GO:0019684), ‘photosynthesis, light harvesting’ (GO:0009765), ‘photosynthesis, light harvesting in photosystem I’ (GO:0009768), and multiple chloroplast and photosystem I/II components, whereas these terms were not significantly affected in Fern−like leaves (**Supplementary Table 2**). In addition, both groups showed enrichment of the BP term ‘response to stress’ (GO:0006950), while downregulated DEGs in Shoestring were specifically enriched for the Cellular Component (CC) terms ‘cell wall’ (GO:0005618), ‘external encapsulating structure’ (GO:0030312), and ‘plasmodesmata’ (GO:0009506) consistent with altered cell wall architecture and symplastic connectivity, which tobamoviral movement proteins can modulate by affecting callose deposition and plasmodesmata−associated proteins during cell−to−cell spread (Sheshukova et al., 2020). By contrast, Fern-like leaves did not show enrichment of photosynthesis−related terms, but instead upregulation of the BP terms ‘lipid biosynthetic process’ (GO:0008610) and ‘lipid metabolic process’ (GO:0006629), which is associated with induction of defense responses (Seth et al., 2024), together with ‘lipid oxidation’ (GO:0034440), suggesting activation of fatty acid oxygenation and production of oxylipins and other oxygenated lipids that act as defense−related signaling molecules during biotic stress (Pretorius et al., 2021).

### KEGG pathway enrichment analysis revealed MAPK signaling in Shoestring and defense−related metabolism in Fern−like leaves

KEGG pathway enrichment analysis of DEGs in the three phenotypic groups showed that Shoestring and Mosaic leaves shared significant enrichment of downregulated DEGs pathways related to ‘photosynthesis’ (sly00195), ‘photosynthesis – antenna proteins’ (sly00196) and ‘porphyrin and chlorophyll metabolism’ (sly00860), whereas these pathways were not enriched in Fern-like leaves, mirroring the GO enrichment analysis (**Supplementary Table 3** – KEGG pathway enrichment analysis). In Shoestring leaves, KEGG analysis of upregulated DEGs identified ‘MAPK signaling pathway – plant’ (sly04016) as enriched (23 genes) whereas this pathway was not significantly enriched in Fern−like or Mosaic leaves. The enriched ‘MAPK signaling pathway – plant’ term in Shoestring included canonical MAPK cascade components (mitogen-activated protein kinase 3, *MPK3*; MAP kinase kinase 5, *MKK5*; MAP kinase kinase kinase 56, *MAPKKK56*), ethylene pathway components (ethylene receptor homolog (ETR4), *ETR4−like*; ethylene receptor-like protein 6, *ETR6−like*; Ethylene Insensitive 3/EIN3−Like, *EIN3/EIL*; ethylene Insensitive 5/7, *EIN5/7*), WRKY TFs (*WRKY22*, *WRKY33A*, *WRKY33B*) and a SNF1-related kinase protein identified as *SnRK2.8* (Fernández et al., 2025). KEGG enrichment analysis of Fern-like leaves showed enrichment of ‘biosynthesis of secondary metabolites’ (sly01110), ‘sesquiterpenoid and triterpenoid biosynthesis’ (sly00909), ‘fatty acid metabolism’ (sly01212) and ‘fatty acid degradation’ (sly00071). Fern−like leaves also showed enrichment of ‘peroxisome’ (sly04146), ‘valine, leucine and isoleucine degradation’ (sly00280), and ‘sulfur metabolism’ (sly00920), in line with enhanced peroxisomal β−oxidation and precursor supply for JA and other lipid−derived defense metabolites (Koo and Howe, 2007).

### Contrasting auxin signaling patterns align with Shoestring and Fern-like leaf manifestations

**Figure 5:**
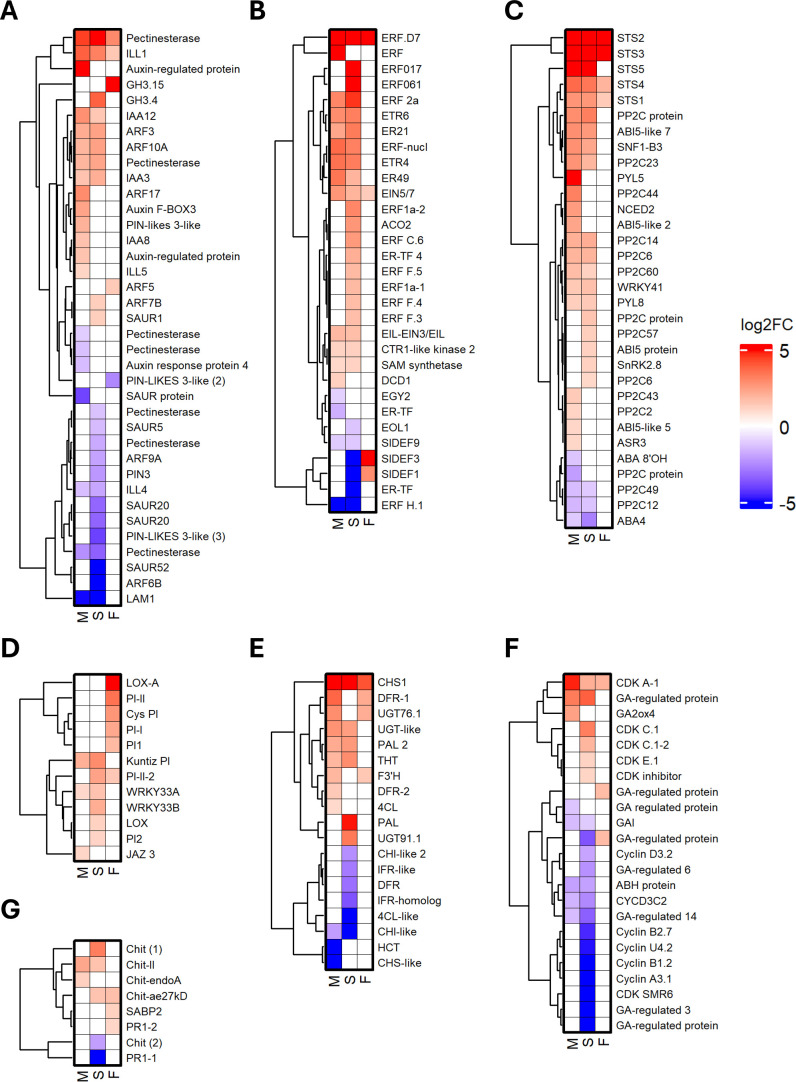
Heatmap visualizations of differentially expressed genes associated with modified hormonal signaling in ToBRFV-A134T- and ToBRFV-WT-infected plants compared to healthy controls. **(A)** Auxin-associated differentially expressed genes (DEGs); **(B)** Ethylene-associated DEGs; **(C)** Abscisic acid (ABA)-associated DEGs; **(D)** Jasmonic acid (JA)-associated DEGs; **(E)** Phenylpropanoid pathway associated DEGs; **(F)** Gibberellic acid (GA)-associated DEGs; **(G)** Salicylic acid (SA)-associated DEGs. Color scale represents log2fold change. Empty cells represent genes with p-value > 0.05 or log2fold change between -1 and 1.

Analysis of auxin-responsive and auxin-related DEGs showed upregulation of multiple ARFs, Aux/IAA proteins and pectinesterases in Mosaic leaves (**Figure 5A**; **Supplementary Table 4** – Pathway-associated DEGs). In comparison, Shoestring leaves exhibited a more divergent expression trend, with upregulation of key auxin signaling components (including *ARF3*, *ARF6b*, *ARF10A*, *IAA3* and *IAA12*) together with strong downregulation of multiple SAUR genes, selected ARFs and pectinesterases. An auxin efflux carrier gene *PIN3*, a PIN family protein involved in polar auxin transport, was strongly downregulated, while the auxin conjugating *GH3.4* (Gretchen Hagen 3.4) was upregulated, indicating adjustments to auxin homeostasis. Additionally, RT-qPCR analysis of *SAUR1* expression showed strong Shoestring-specific induction consistent with RNA-seq data (**Figure 5B**). In Fern-like leaves, the number of auxin-responsive DEGs was markedly lower than in both Mosaic and Shoestring leaves. *GH3.15*, an IAA-conjugating enzyme that reduces free auxin levels in tomato (Ai et al., 2023), was strongly upregulated uniquely in Fern-like leaves. Notably, three auxin-related regulators with established roles in leaf morphogenesis showed symptom-specific regulation. *LAM1*, a WOX (WUSCHEL−related homeobox) family gene required for leaf blade expansion and secondary leaflet formation in tomato, was strongly downregulated in Shoestring and Mosaic leaves (Wang et al., 2021). Consistent with its upregulation in early Shoestring manifestations (Molad et al., 2025), *ARF3* was upregulated in both Shoestring and Mosaic leaves. In contrast, *ARF5*, which promotes auxin-dependent leaflet formation and leaf blade expansion in tomato, was specifically upregulated in Fern-like leaves (Israeli et al., 2019; Wójcikowska et al., 2023). Together, *LAM1*/*ARF3* in Shoestring/Mosaic, and *ARF5* in Fern-like leaves, highlight the central role of auxin in shaping each symptom manifestation.

### Shoestring leaves exhibit strong ethylene pathway activation while ethylene-induced defense genes are downregulated

**Figure 6:**
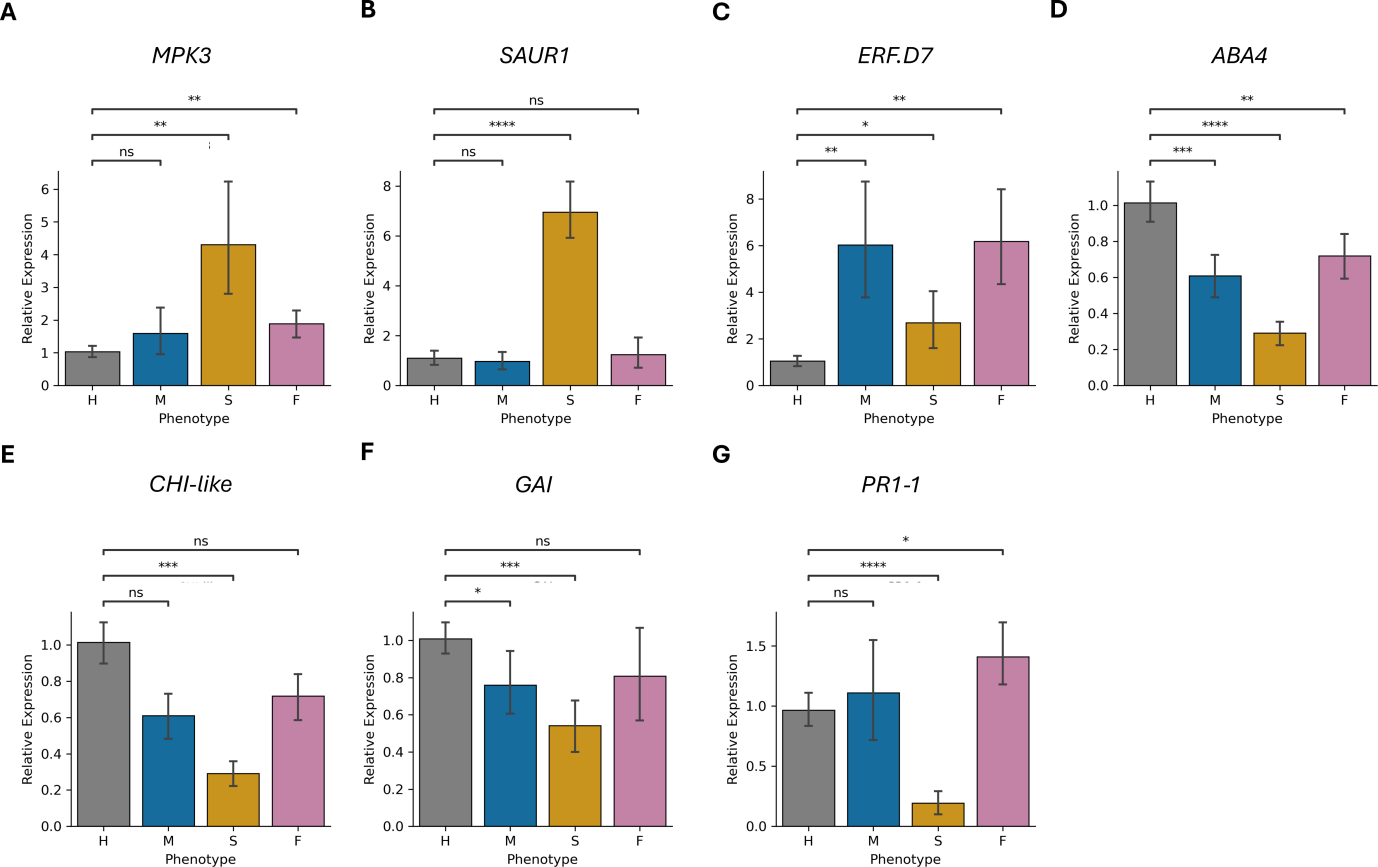
RT-qPCR analyses of selected differentially expressed genes. Y-axis represents relative expression, measured by 2^(−ΔΔ*CT*) mean expression, X-axis represents phenotypes (H, Healthy; M, Mosaic; S, Shoestring; F, Fern-like). Error bars indicate 95% confidence interval of biological replicates. Asterisks denote statistical significance (* - p-value < 0.05, ** - p-v0.001, *** - p-value < 0.001, **** - p-value < 0.0001). **(A)** MPK3, Mitogen-activated protein kinase 3; Solyc06g005170; **(B)** SAUR, Small auxin up-regulated RNA1; Solyc01g091030; **(C)** ERF.D7, Ethylene responsive transcription factor D7; Solyc03g118190; **(D)** ABA4, Abscisic acid deficient 4; Solyc02g086050; **(E)** CHI, Chalcone-flavanone isomerase family protein; Solyc02g083890; **(F)** GAI, Gibberellic acid insensitive; Solyc11g011260; **(G)** PR1-1, Pathogenesis-related protein PR-1; Solyc07g006710.

Analysis of ethylene (ET)-responsive genes showed strong upregulation of a large subset of transcription factors (ERFs/ER-TFs) in Shoestring leaves, with partially overlapping induction patterns in Mosaic leaves (**Figure 5B**; **Supplementary Table 4**). In both groups, key components of ethylene biosynthesis and signaling were upregulated, namely *EIN3/EIL*, *EIN5/7*, S−adenosyl−L−methionine synthetase (SAM synthetase) and a CTR1−like protein kinase 2 gene (Cho and Yoo, 2015). A 1−aminocyclopropane−1−carboxylate oxidase 2 (*ACO2*) gene, involved in ethylene biosynthesis (Barry et al., 1996), was uniquely and significantly upregulated in Shoestring leaves. Notably, both groups show very strong downregulation of Ethylene Response Factor H.1 (*ERF H.1*), which in healthy tomato seedlings was shown to be induced by both ethylene and auxin (Pirrello et al., 2012). Three Defensin genes - *DEF1*, *DEF3* and *DEF9*, encoding antimicrobial peptides associated with anti-fungal defense responses (Nikoloudakis et al., 2020), were strongly downregulated in Shoestring leaves, whereas two of these genes are upregulated in Fern-like leaves, despite the near absence of broad ERF induction. RT-qPCR analysis of *ERF.D7*, a positive regulator of auxin and ethylene signaling pathways in tomato fruit (Gambhir et al., 2022), showed statistically significant upregulation in all phenotype groups, albeit lower in Shoestring leaves (**Figure 6C**).

### Altered ABA biosynthesis and signaling components separate Shoestring from Mosaic and Fern-like leaves

Analysis of ABA-associated DEGs revealed strong upregulation of five sesquiterpene synthase (STS) genes in Shoestring and Mosaic leaves (**Figure 5C**; **Supplementary Table 4**). In Fern-like leaves, four out of five STSs were upregulated as well, but all of them had lower log2 fold−change values compared to both Shoestring and Mosaic leaves. The tomato abscisic acid−deficient 4 (*ABA4*) gene, encoding a neoxanthin biosynthesis protein required for ABA production and the *Arabidopsis ABA4* ortholog, was strongly downregulated in Shoestring leaves and moderately downregulated in Mosaic leaves in the DEG analysis data leaves (Neuman et al., 2014; Perreau et al., 2020) (**Figure 5C**). RT−qPCR analysis supported the DEG analysis results by confirming *ABA4* downregulation in Shoestring and Mosaic leaves and notably showed statistically significant downregulation in Fern−like leaves (**Figure 6D**). The *NCED2* gene, encoding a 9−cis−epoxycarotenoid dioxygenase that catalyzes the key carotenoid−cleavage step in ABA biosynthesis and belongs to the NCED clade closely related to *Arabidopsis AtNCED3* (Holsteens et al., 2022), was upregulated strictly in Mosaic leaves. The ABA 8’-hydroxylase (*ABA8’OH*) gene, which initiates ABA 8’-hydroxylation and thereby a major route for ABA catabolism (Krochko et al., 1998), was downregulated solely in Mosaic leaves. Multiple protein phosphatase 2C (PP2C) genes were also differentially expressed in Shoestring and Mosaic leaves, forming distinct up- and downregulated subsets in each of the two phenotype groups, while *SnRK2.8* was upregulated exclusively in Shoestring as part of the enriched MAP kinase pathway (**Figure 5C**; **Supplementary Table 3**).

### Divergent JA−responsive defense programs distinguish Shoestring and Fern-like manifestations

Analysis of jasmonic acid (JA)-associated DEGs revealed induction of multiple JA-responsive proteinase inhibitor and transcription factor genes in all three phenotype groups, with Mosaic leaves showing the fewest DEGs and Shoestring showing almost no DEG overlap with Fern-like leaves (**Figure 5D**; **Supplementary Table 4**). In Shoestring leaves, several JA−associated defense genes were significantly upregulated, including proteinase inhibitor 2 (*PI2*), a Kunitz−type protease inhibitor, Proteinase inhibitor type-2 (*PI−II−2*), a lipoxygenase (*LOX*) and two WRKY transcription factors. The two WRKY TFs upregulated in Shoestring leaves correspond to the two tomato homologues of Arabidopsis *WRKY33*, implicated in JA/Et-mediated defense against necrotrophic pathogens (Zhou et al., 2015). In Fern−like leaves, JA-associated DEGs included strong upregulation of Lipoxygenase A (*LOXA*), multiple proteinase inhibitor genes and a cysteine proteinase inhibitor (Cys PI), consistent with the GO enrichment of lipid metabolic, lipid biosynthetic and lipid oxidation (**Figure 5D**; **Supplementary Table 2**).

### The flavonoid branch of the phenylpropanoid pathway is repressed in Shoestring leaves

Analysis of DEGs associated with the phenylpropanoid pathway revealed significant upregulation of two phenylalanine ammonia-lyase (*PAL*) genes we termed *PAL* and *PAL2* in Shoestring leaves (**Figure 5E**; **Supplementary Table 4**). However, several key genes downstream were strongly downregulated, including chalcone isomerase−like genes (*CHI−like* and *CHI−like 2*), dihydroflavonol 4−reductase (*DFR*), isoflavone reductase−like (*IFR−like*), an IFR−homolog and a 4−coumarate−CoA ligase−like (*4CL−like*) gene. This pattern indicates a bottleneck in the flavonoid branch of the phenylpropanoid pathway, a branch widely associated with induced flavonoid biosynthesis and plant defense responses (Ramaroson et al., 2022). In Mosaic leaves, suppression of the flavonoid branch was limited to a *CHI-like* gene and a *CHS-like* gene, along with downregulation of a gene identified as shikimate hydroxycinnamoyl transferase (*HCT*), associated with the lignin branch (Rigano et al., 2016) (**Figure 5E**). RT-qPCR analysis of this *CHI-like* gene confirmed its downregulation in Shoestring with no statistical significance in Mosaic and Fern-like leaves (**Figure 6E**). In Fern-like leaves, *CHS1* and several late flavonoid enzymes, including flavonoid-3′-hydroxylase (*F3’H*) and flavanone reductase (*DFR*) family members, were significantly upregulated, with no detected downregulation of genes in the flavonoid branch.

### Shoestring leaves exhibit strong downregulation of GA-related cell division cyclins

Gibberellic-Acid Insensitive (*GAI*), a DELLA family growth repressor that negatively regulates GA signaling (Wang et al., 2020), was downregulated in both Mosaic and Shoestring leaves (**Figure 5F**; **Supplementary Table 4**). RT-qPCR analysis confirmed statistically significant *GAI* downregulation in both groups (**Figure 6F**). Despite GAI downregulation, DEG patterns support overall GA pathway attenuation in both phenotype groups. In Mosaic leaves, Gibberellin 2−oxidase 4 (*GA2ox4*), which catalyzes GA deactivation (Chen et al., 2016), was upregulated, while several GA-regulated genes were downregulated. In Shoestring leaves, downregulation of GA-induced genes was apparent, including strong suppression of cyclin genes (*CycA3.1*, *CycB1.2*, *CycB2.7*, *CycU4.2*, *CycD3.2*, *CycD3C2*), indicating suppression of cell division (**Figure 6F**).

### Salicylic acid−associated genes showed opposite expression trends in Shoestring and Fern−like leaves

Analysis of SA-associated DEGs showed that Shoestring and Fern-like leaves exhibited opposite expression patterns of two closely related Pathogenesis-related protein 1 (*PR-1*) genes we termed *PR1–1* and *PR1-2* (**Figure 5F**; **Supplementary Table 4**). In Shoestring leaves, *PR1–1* was strongly downregulated, and PR1–2 was not differentially expressed, whereas in Fern-like leaves *PR1–2* was upregulated (**Figure 5F**). In Mosaic leaves neither of the PR-1 genes were differentially expressed. RT-qPCR analysis confirmed statistically significant downregulation of *PR1–1* in Shoestring leaves and upregulation in Fern-like leaves (**Figure 6G**). Both PR-1 genes encode CAP/PR-1 family pathogenesis-related proteins that belong to the same tomato *PR-1* subgroup and are generally associated with SA-linked defense (Akbudak et al., 2020). Furthermore, salicylic acid-binding protein 2 (*SABP2*), which converts methyl salicylate to SA and is involved in SAR development and *PR-1* upregulation in response to TMV infection in tobacco plants (Kumar and Klessig, 2003), was significantly upregulated only in Fern-like leaves.

### ToBRFV accumulation is not associated with symptom manifestation

**Figure 7:**
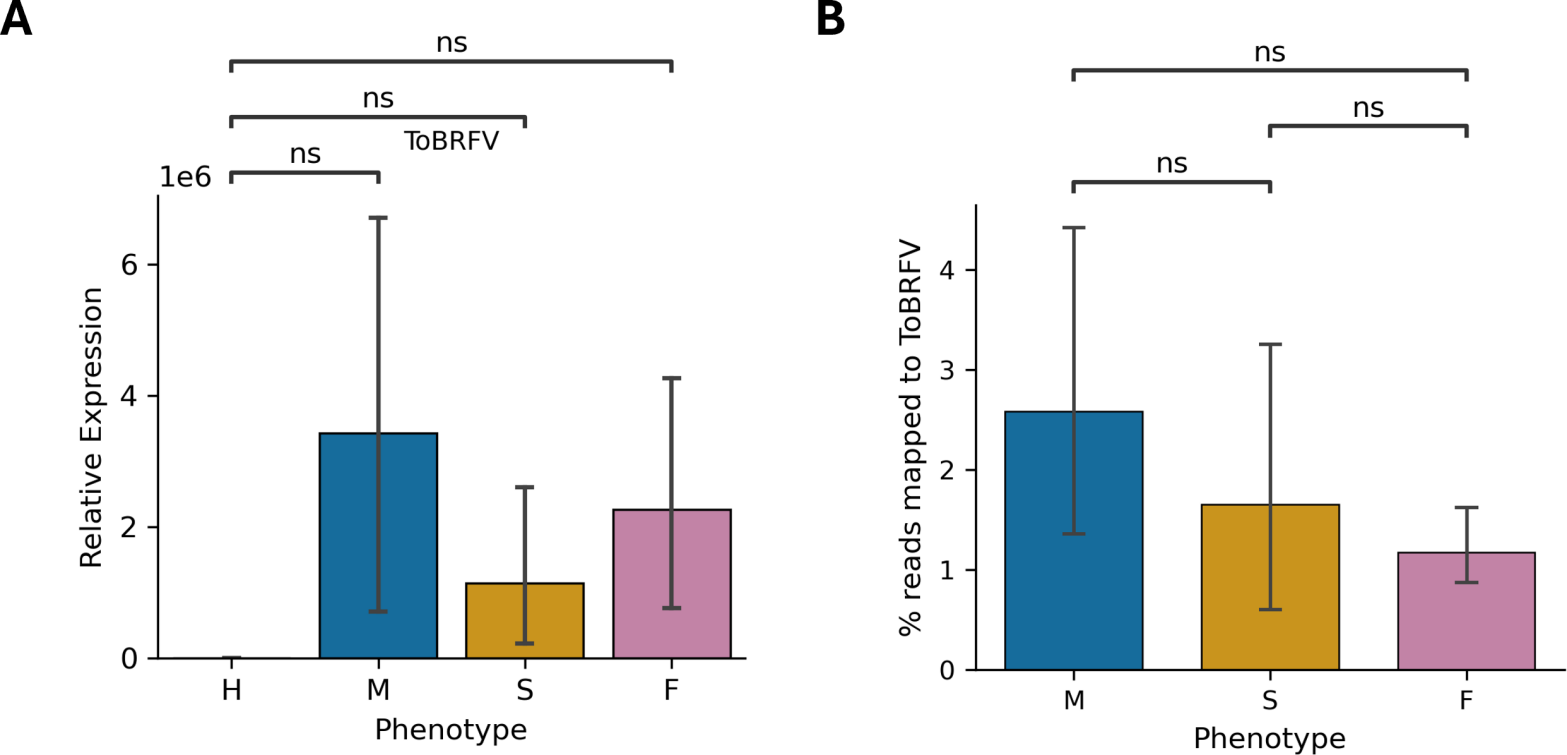
ToBRFV accumulation in tomato leaves exhibiting mosaic, shoestring and fern-like symptoms. **(A)** RT-qPCR analysis of ToBRFV coat protein expression in tomato leaves. Y-axis represents relative expression measured by 2^(−ΔΔC~T~) mean expression. Error bars indicate 95% confidence interval of biological replicates. **(B)** Percentage of RNA-seq reads mapped to the ToBRFV genome per biological replicate. Error bars indicate 95% confidence interval of biological replicates. In both panels, X-axis represents phenotypes (M, Mosaic; S, Shoestring; F, Fern-like) and bars marked “ns” indicate no statistically significant difference between groups (Welch’s t-test, p > 0.05).

To assess whether the transcriptional differences observed between the different symptoms could be attributed to differential viral accumulation rather than specific gene expression, ToBRFV accumulation was quantified across all sampled leaf manifestations. RT-qPCR analysis of ToBRFV accumulation showed substantial variability among biological replicates within each phenotype group, with no statistically significant differences between Mosaic, Shoestring and Fern-like leaves (**Figure 7A**). Examination of RNA-seq read counts mapped to the ToBRFV reference genome showed comparable percentages across all three phenotypes, ranging from 0.61% to 4.42%, with overlapping ranges and no statistically significant differences between groups (**Figure 7B**). Notably, Fern-like leaves – despite their transcriptional co-clustering with Healthy leaves – contained substantial ToBRFV accumulation confirmed both by RT-qPCR and RNA-seq read mapping, confirming that their transcriptional state is not directly associated with reduced viral load.

## Discussion

ToBRFV-A134T is a naturally occurring isolate which induces severe shoestring-like and fern-like foliar manifestations in *Tm-2^2^*-harboring tomato plants (Molad et al., 2025). In this study we show that severe shoestring symptoms consistently precede fern-like symptoms, and both manifestations are coupled with transcriptional reprogramming of auxin and other phytohormone-associated genes that affect tomato leaf formation and shape. Tomato plants infected with ToBRFV-WT do not exhibit severe shoestring or fern-like symptoms but can exhibit mild shoestring symptoms (**Figures 1A3**, **2**). Exogenous treatments of auxin increased the frequency of shoestring manifestations in ToBRFV-WT infected tomatoes and induced fern-like symptoms, without the A134T mutation background, effectively recapitulating the chronological symptom development of ToBRFV-A134T infection (**Figure 3**). These results strengthen our previous findings that auxin plays a role in shoestring manifestations in the context of ToBRFV infection and in the transition to a fern-like state (Molad et al., 2025).

Despite shoestring and fern-like manifestations being distinct sequential phases, at later disease stages ToBRFV-A134T-infected plants exhibit a mixture of these phenotypes together with characteristic mosaic symptoms (**Figures 1C4**, **2**; **Supplementary Figure 1**). This phenomenon informed our sampling strategy, allowing for a clear transcriptomic comparison of mosaic leaves from ToBRFV-WT infected plants and both shoestring and fern-like leaves from ToBRFV-A134T infected plants, with asymptomatic leaves from non-infected tomato plants. At the transcriptional level, A134T-induced shoestring leaves showed the largest deviation from healthy controls, clustering with ToBRFV-WT mosaic leaves, whereas fern-like leaves clustered with healthy controls (**Figure 4C**). Shoestring leaves shared a much larger overlap of DEGs with WT-induced mosaic leaves than with A134T-induced fern-like leaves, despite the difference in isolate used as inoculum (**Figures 4A, B**). This data suggests that while shoestring and fern-like leaves are a consistent part of A134T symptomatology, they are vastly different states of disease, with fern-like leaves representing a state of temporary partial recovery. The co-clustering with healthy controls is consistent with a partial restoration of basal transcriptional programs rather than an absence of infection, as evidenced by substantial ToBRFV accumulation confirmed both by RT-qPCR and RNA-seq read mapping (**Figure 7A**, **Supplementary Figure 2**).

Analysis of pathway-associated DEGs indicates that auxin could be a key driver of A134T symptomatology specifically while also engaged during ToBRFV infection in tomato generally. ToBRFV-WT-induced mosaic leaves exhibit upregulation of multiple ARFs, Aux/IAA genes and pectinesterases as part of an auxin-responsive program, consistent with reports that some plant viruses can reprogram auxin signaling and Aux/IAA–ARF modules to enhance infection, including the model tobamovirus TMV (**Figure 5A**) (Collum et al., 2016; Padmanabhan et al., 2008; Vinutha et al., 2020). In shoestring leaves, auxin signaling rewiring leads to both up- and downregulation of ARFs, Aux/IAA, pectinesterase and SAUR proteins (**Figure 5A**). Upregulation of *GH3.4* and downregulation of *PIN3* indicate altered auxin transport and conjugation, and the downregulation of *LAM1*, a WOX family gene whose loss in tomato causes narrow leaves with reduced secondary leaflets, further illustrates auxin influence on A134T-induced symptomatology (Wang et al., 2021). This combination supports a scenario in which ToBRFV-A134T infection induces a non-canonical auxin regime that disrupts normal lamina growth and venation, contributing to the narrow, wiry-like morphology (Kazan and Manners, 2009; Yifhar et al., 2012). These patterns are consistent with reports that plant viruses, including the model tobamovirus TMV, can reprogram auxin signaling to enhance infection, and that elevated auxin responses can antagonize SA-dependent defenses (Manulis et al., 1998; Padmanabhan et al., 2008, 2005), as discussed in the introduction. In fern-like leaves, by contrast, the auxin signature is muted and the number of auxin-responsive DEGs is considerably reduced (**Figure 5A**). *GH3.15*, an IAA-conjugating enzyme implicated in lowering free auxin levels, and *ARF5*, associated with auxin-dependent organ growth and leaf patterning, are uniquely upregulated as part of a compensatory response to increase the leaf blade area (Ai et al., 2023; Wójcikowska et al., 2023).

ET-, JA- and ABA-associated pathways further differentiate shoestring from fern-like leaves and highlight extensive hormonal convergence with synergistic, additive and antagonistic interactions among defense and growth pathways (Pan et al., 2021). Both shoestring and mosaic leaves exhibit activation of core ET biosynthesis and signaling components, including *EIN3/EIL*, *EIN57*, *SAM* and a *CTR1*-like kinase, indicating robust ET pathway activation, with shoestring leaves additionally showing unique upregulation of *ACO2* and a much broader ERF induction (**Figure 5B**). In parallel, *DEF* (Defensin) genes, typically associated with anti-fungal defense, are strongly downregulated in shoestring leaves, whereas in fern-like leaves two of these Defensins are upregulated despite much weaker ERF upregulation. JA-associated DEGs also split along phenotype boundaries: The JA-responsive defense genes including PI2, PIII2, a lipoxygenase and *WRKY33* homologues are upregulated in shoestring leaves, consistent with a necrotroph-like JA/ET defense module, whereas in fern-like leaves *LOXA*, multiple proteinase inhibitors and a cysteine proteinase inhibitor alongside are preferentially induced, alongside GO and KEGG enrichments of lipid metabolism, biosynthesis and oxidation (**Figure 5D**; **Supplementary Tables 2**, **3**). The strong induction of ERFs in shoestring leaves includes components that have been implicated in both defense responses and fruit ripening processes in tomato fruit, such as ERF.C6, ERF.D7, and several F-group ERFs (ERF.F3, ERF.F4, ERF.F5). While the overlap between ripening and defense functions of these genes has been described and studied primarily in fruit, including possible roles in hormone crosstalk (Gambhir et al., 2022; Li et al., 2025, 2022; Liu et al., 2016; Pérez-Llorca et al., 2019), the strong upregulation of these ERFs in leaves in the context of ToBRFV infection warrants further investigation.

ABA-related genes further highlight the similarity between mosaic and shoestring leaves and their distance from fern-like leaves. Sesquiterpene synthases are most strongly induced in shoestring and mosaic leaves, *ABA4* is downregulated in all infected phenotypes, and *SnRK2.8* is uniquely upregulated in shoestring as part of the enriched MAPK pathway, pointing to integration of ABA signaling into the broad hormonal pathway convergence. Collectively, these patterns indicate that shoestring leaves represent a high-convergence zone where auxin, ET, JA and ABA pathways are simultaneously engaged and extensively reprogrammed, while fern-like leaves retain only a subset of these outputs and at lower amplitude (Supplemental **Supplementary Table 4**).

MAPK signaling provides a plausible molecular explanation for the shoestring-fern-like transition, but our data support cautious interpretation. KEGG enrichment analysis identifies the plant MAPK signaling pathway exclusively in shoestring leaves, encompassing canonical cascade components and central components of the active hormonal pathways (**Supplementary Table 3**). RT-qPCR analysis confirmed strong *MPK3* upregulation in shoestring leaves and a weaker yet statistically significant increase in fern-like leaves. These observations are consistent with studies in Arabidopsis and tomato showing that MAPK cascades, including *MPK3*, can amplify or prime defense responses and intersect with hormone pathways such as SA and auxin (Pan et al., 2021). However, because our RNA-seq captures a single point at a late disease stage, we cannot resolve the temporal ordering of transcriptional changes relative to symptom onset more broadly, including the ordering of MAPK activation relative to SA and other hormone changes. Future time-course studies, sampling the early, intermediate and late stages of symptom development, will be necessary to distinguish causal from downstream hormonal and transcriptional responses.

The contrasting phenylpropanoid and GA-related expression programs in shoestring and fern-like leaves reinforce our view of divergent growth/defense tradeoffs in the two phases. In shoestring leaves, two *PAL* genes are strongly induced while multiple key flavonoid-branch enzymes, including *CHI*-like, *DFR*, *IFR*-like and *4CL*-like genes, are downregulated, possibly indicating a bottleneck that limits downstream flavonoid accumulation despite activation of the pathway’s entry point (**Figure 5E**). In mosaic leaves this suppression is more modest, whereas in fern-like leaves *CHS1* and late flavonoid enzymes such as *F3’H* and *DFR* family members are upregulated without detectable downregulation of other genes in the branch, suggesting restoration of flavonoid biosynthesis capacity. GA-associated genes follow a similar pattern: the DELLA repressor *GAI* is downregulated in both shoestring and mosaic leaves, confirmed by RT-qPCR analysis (**Figures 5F**, **6F**). However, shoestring leaves show strong downregulation of multiple cyclin genes indicative of GA-dependent cell cycle suppression, consistent with constrained lamina growth and narrow leaves. Fern-like leaves, in contrast, lack this broad repression of cyclins, in line with their increased leaf area. SA-associated genes also invert between each phase: RT-qPCR analysis showed *PR1–*1 is suppressed in shoestring and induced in fern-like leaves, while DEG analysis revealed *PR1–*2 is uniquely upregulated in fern-like leaves, and the SA-binding protein *SABP2* is induced only in fern-like leaves as well (**Figure 5G**). These patterns point to active SA-mediated defense in fern-like leaves, in contrast to a largely SA-suppressed state in shoestring leaves and supports our partial-recovery hypothesis. The apparent re-activation of SA-mediated defense in fern-like leaves is consistent with reports that SA accumulation promotes resistance to tobamoviruses and that SA and auxin engage in antagonistic crosstalk, where high auxin suppresses SA signaling and vice versa (Venturuzzi et al., 2021; Wang et al., 2007).

Our exogenous auxin application experiments provide additional support for the role of auxin-associated processes in shaping the shoestring and fern-like trajectory. Application of the synthetic auxin NAA to ToBRFV-WT-infected plants recapitulates the characteristic sequence of severe shoestring followed by fern-like leaves, without inducing comparable symptoms in mock-inoculated or NAA-only plants (**Figure 3**). These results indicate that an augmented auxin response in the context of ToBRFV infection can contribute to manifesting the same symptoms induced by A134T-infected tomato plants. When placed alongside the RNA-seq and RT-qPCR data, the auxin treatments support a model in which ToBRFV-A134T shifts auxin signaling and MAPK activity into a regime associated with a high-energy, multi-hormone convergence state characteristic of shoestring leaves. These leaves subsequently transition into a lower−intensity, SA−competent fern-like state, with a transcriptome closer to healthy, asymptomatic leaves. In this model, shoestring leaves represent the phenotypical consequence of extensive crosstalk between auxin and classical defense hormones, where auxin can antagonize SA-dependent defenses yet cooperate with JA-associated responses depending on context, combined with growth/defense tradeoffs governed by GA, ABA and other regulators (Kazan and Manners, 2009; Pan et al., 2021; Zhao and Li, 2021).

**Figure 8:**
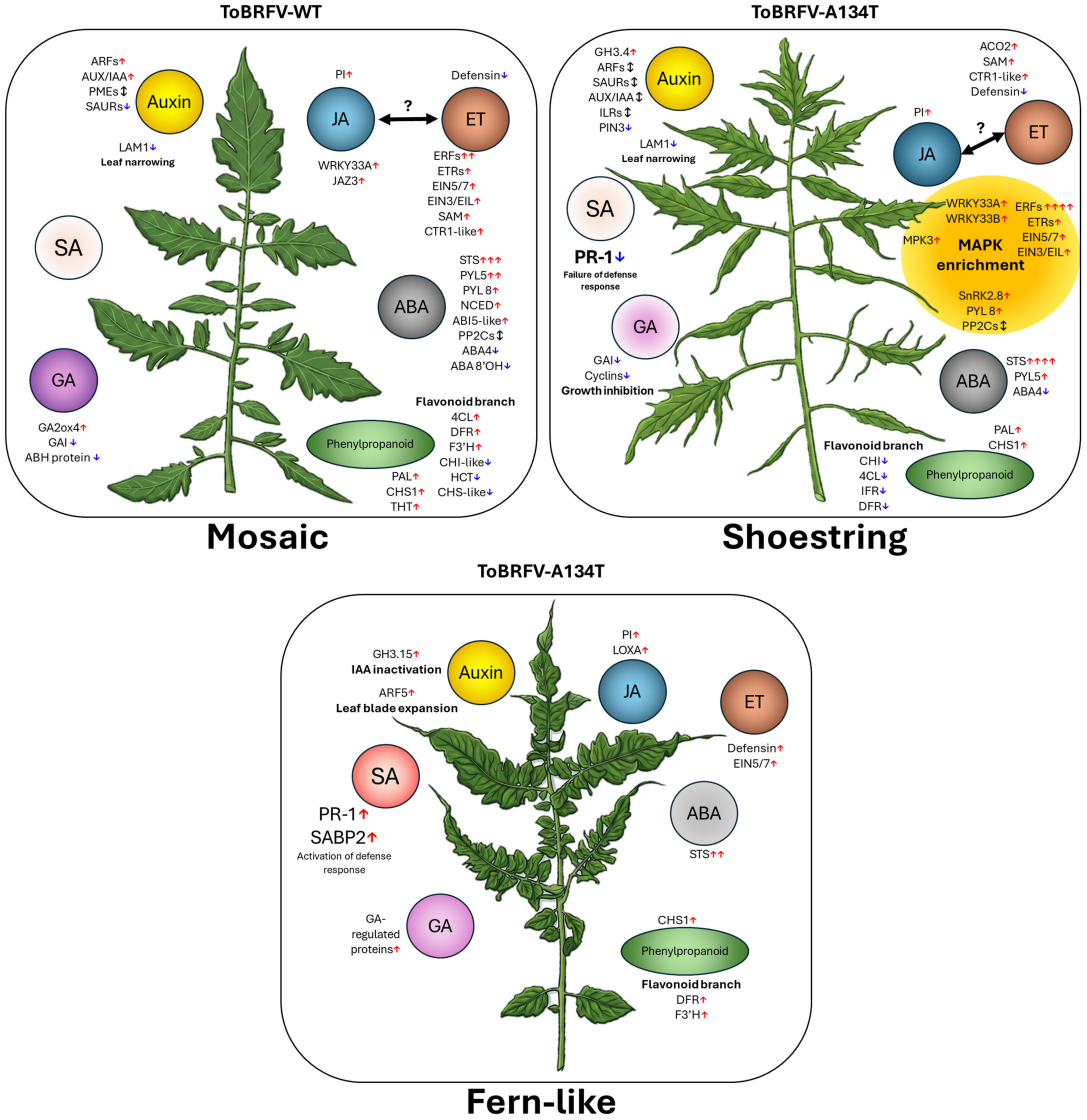
Schematic representation of pathway reprogramming in ToBRFV−infected tomato plants displaying the Mosaic, Shoestring, and Fern−like phenotypes. Colored circles indicate hormone pathways (Auxin, JA, ET, SA, GA, ABA) and the green oval indicates the phenylpropanoid pathway; red and blue arrows denote transcriptional up− and down−regulation of representative genes, respectively, based on RNA−seq differential expression. Only selected, significantly regulated genes are shown; complete DEG lists are provided in **Supplementary Table 1**, and pathway-associated DEG lists are provided in **Supplementary Table 4**).

Altogether, our data support a phase-based view of ToBRFV-A134T disease in *Tm-2^2^* -harboring tomato plants, where unique manifestations correspond to contrasting transcriptional and hormonal states (**Figure 8**). Severe shoestring leaves coincide with extensive auxin pathway rewiring, MAPK enrichment, strong ET/JA/ABA engagement, repression of SA and bottlenecked flavonoid biosynthesis, alongside suppression of GA-associated growth modules. Fern-like leaves, by contrast, show restoration of SA-linked defense, a limited auxin signature and absence of MAPK pathway enrichment, yielding a temporary, partial recovery-like state until the reemergence of typical disease symptoms. The ability of exogenous auxin to recapitulate the shoestring–fern-like sequence in ToBRFV-WT infection is consistent with auxin-associated processes being a key entry point into this trajectory and suggests that the A134T movement protein mutation may act, at least in part, by reshaping auxin–MAPK–defense crosstalk. Future work will need to shed light on the specific influence of the A134T mutation on MP-plant interactions that could induce this differential transcriptional rewiring.

## Data Availability

The datasets presented in this study can be found in online repositories. Transcriptome data used in this paper has been deposited in Sequence Read Archive (SRA) repository, BioProject: PRJNA1431405.
