# Supplementary figures and images for: Rewiring of auxin and MAPK signaling is associated with contrasting shoestring and fern-like manifestations in ToBRFV-A134T-infected tomato
Source: Front Plant Sci. 2026 Jun 30;17:1828330. doi: 10.3389/fpls.2026.1828330 (PMC13364930; doi:10.3389/fpls.2026.1828330)

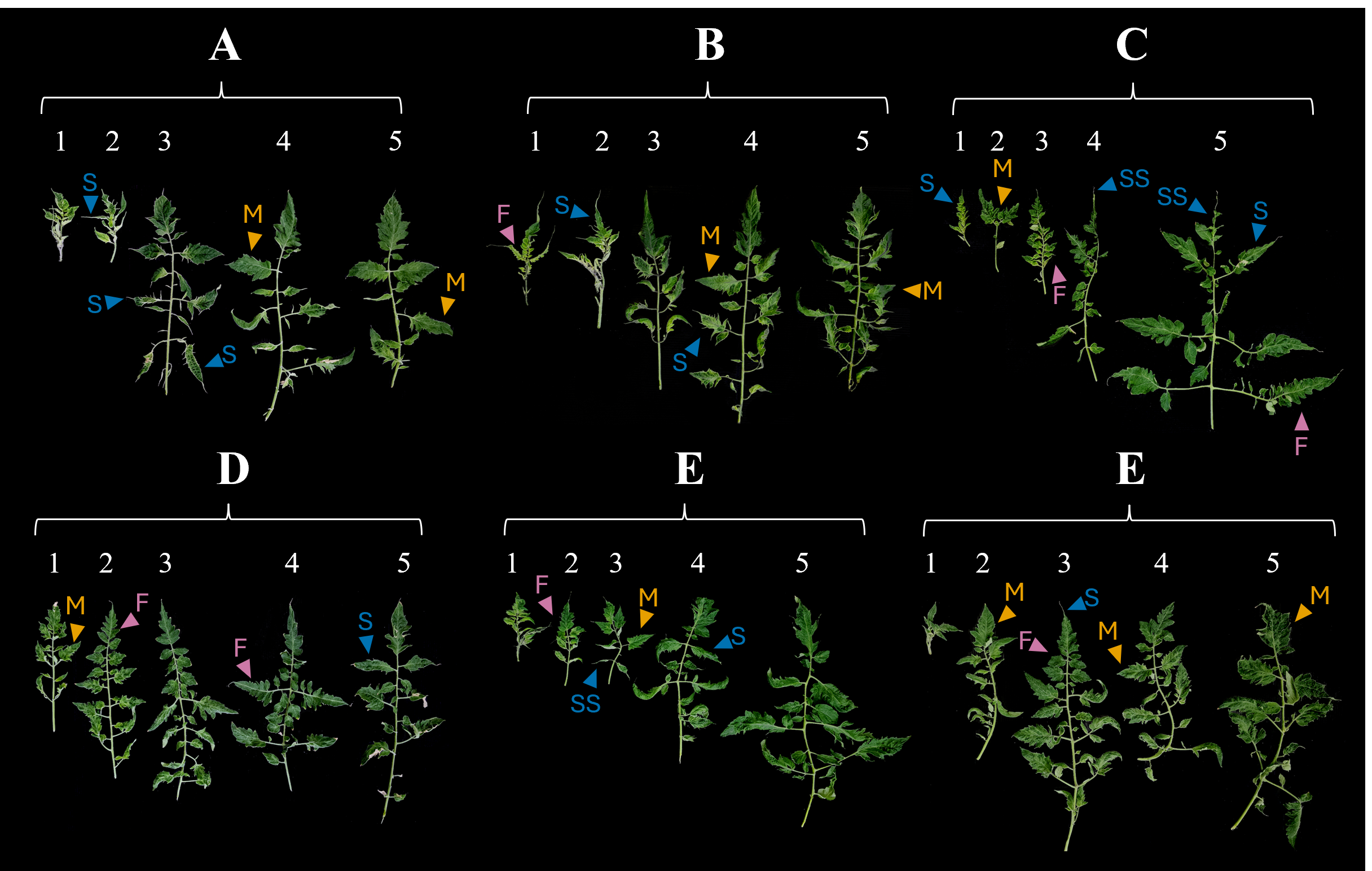

Supplement: Supplementary file 1 [file Image1.tif]

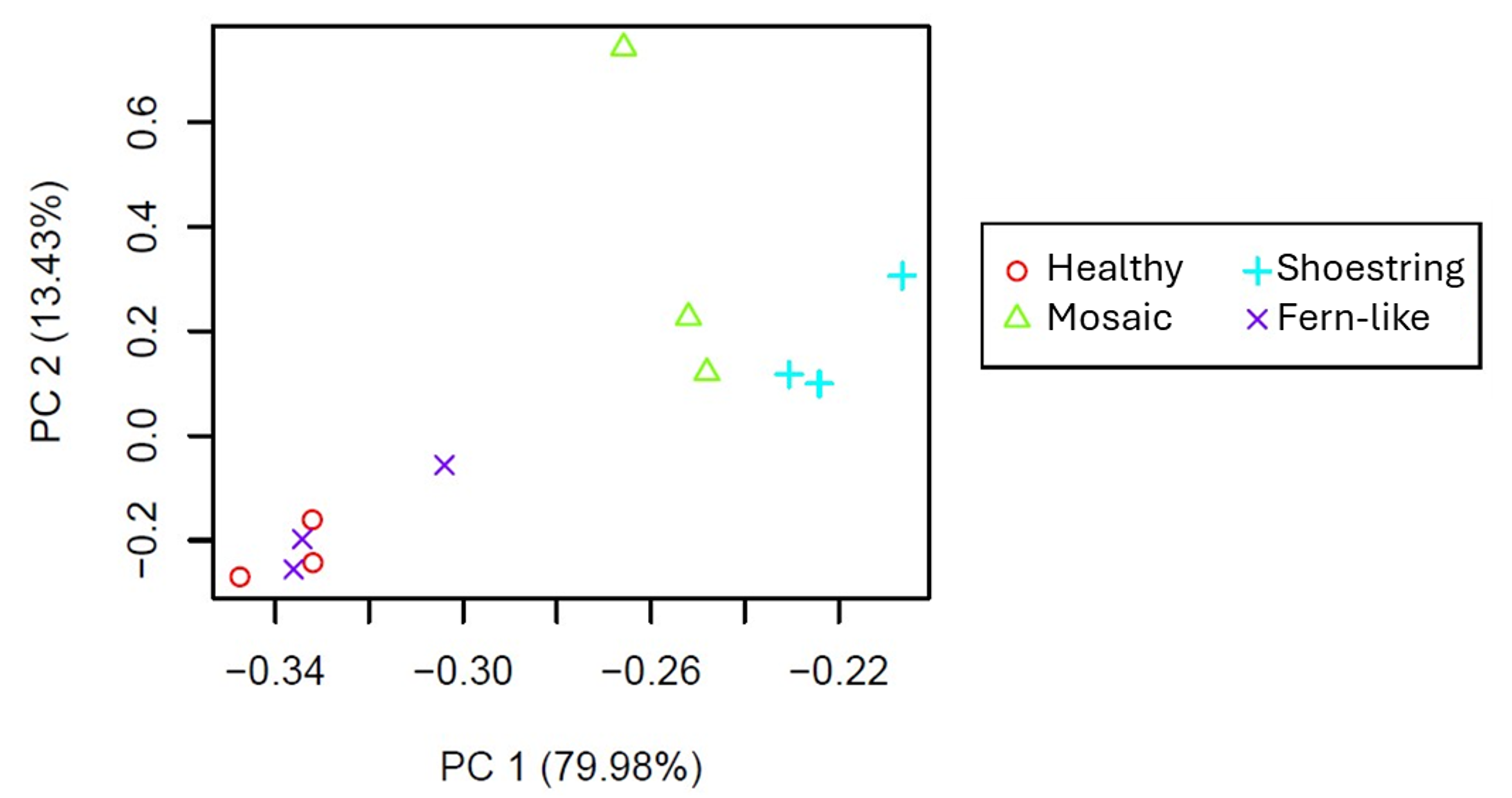

Supplement: Supplementary file 2 [file Image2.tif]
